# Interrogator intonation and memory encoding performance

**DOI:** 10.1371/journal.pone.0218331

**Published:** 2019-06-13

**Authors:** Silvia Gubi-Kelm, Alexander F. Schmidt

**Affiliations:** 1 Department of Psychology, Medical School Hamburg, Hamburg, Germany; 2 Institute of Psychology, Social and Legal Psychology, University of Mainz, Mainz, Germany; Victoria University of Wellington, NEW ZEALAND

## Abstract

Based on recent findings that interrogator intonation can enhance interrogative suggestibility during recall phases, the present study tested influences of interrogator intonation on memory performance even as early as at the encoding stage. We experimentally manipulated interrogator intonation during encoding of a story to be recalled in immediate and delayed subsequent memory tests (Experiment 1, *N* = 50). As expected, a symmetrically structuring vs. an isolating-emphasizing speaking style generally increased the amount of freely recalled details. In a more fine-grained experiment (*N* = 50), we additionally manipulated emphasized story details and tested recall rates for peripheral, neutral, and central items. We found that emphasized peripheral details of the story were easier reproduced than central details realized in a neutral fashion, whereas the opposite pattern emerged for emphasized central details. Results are discussed in terms of their implications for forensic (interrogation) contexts and their legal psychological relevance.

## Introduction

Testimonies in forensic contexts are the result of the interaction between the reproduction efforts of the person answering questions, on the one hand, and the interrogative conduct of the person asking questions, on the other hand. The psychologist William Stern [1] expressed this as early as 1904, when he described a statement as a mental achievement and product of interrogation. As in all conversational situations, the communicative exchange between the interrogator and the interrogee in forensic settings cannot be reduced to verbal content exclusively. It also comprises communicative signals such as, for example, facial expressions, and gestures that accompany speech as well as communicative signals transported via the prosodic features of spoken language and, specifically, its intonation. Jones [2] defines intonation as “the variations which take place in the pitch of the voice in connected speech” (p. 275). Accordingly, from a legal psychological perspective, it has been only recently demonstrated that phrase-falling intonations (indicating claims/facts rather than questions) on the interrogator’s side contributed to increased interrogative suggestibility (i.e. “the extent to which, within a closed social interaction, people come to accept messages communicated during formal questioning, as the result of which their subsequent behavioral response is affected”[3], p. 84) in interviewed participants during the recall phase of more complex verbal information [4]. However, it remains an open empirical question whether the influence of intonation comes into effect as early as in the encoding phase of verbally presented episodes. Such possible early memory alteration effects due to interrogator intonation artifacts should not only be important particularly to research on interrogative suggestibility but forensic practice as well (e.g. during interviews of suspects or witnesses).

Specifically, on the one hand, *direct interrogative suggestibility effects* are possible. At least in German police contexts, it is common practice to read out résumés of larger parts of witness or suspect accounts to the interrogee in order to be verified by her/him. It is thus conceivable that an interrogee predominantly remembers those details which were given auditive salience, and hence significance in the read-out résumé. In principle this is unproblematic, however, once confirmatory hypothesis testing [5] has (involuntarily or deliberately) effected the interrogator’s summary of the interrogated account in question, it might exert a suggestive potential.

On the other hand, *indirect interrogative suggestibility effects* could be hypothesized for the Gudjonsson Suggestibility Scales (GSS-1/GSS-2; [6–9]) procedure on which not only the results of many empirical studies, but also the practical evaluation of testimonies are based [10–12]. The GSS have been translated into different languages and are commonly used as a measurement of individual differences in the vulnerability for interrogative suggestibility [13], namely the tendency to give in to leading questions (Yield) and the tendency to shift responses under conditions of interpersonal pressure (Shift). In short, the GSS consists of a narrative paragraph that is read out or played from an audio tape to the respondent, who then freely reports all she/he recalls about the story. Subsequently, after a delay of 50 minutes, the interrogee freely recalls the story again and is asked a number of questions about the story, most of them are (mis)leading. Next, the interviewee is told that she/he has made a number of errors and needs to answer the questions again. Changes between the different measurement times are regarded as indicators of susceptibility to interrogative suggestibility.

Based on these notions, it is conceivable that the influence of intonation might be extended even before the actual interrogation process [4]. After all, the presentation of the story (at least in case of the GSS) or an interrogees’ prior testimony is already a part of the communicative exchange between interrogator and interrogee. It thus seems possible that the prosodic constituents of the verbal information already come into effect in early encoding stages thereby influencing the later reproduction/recall phase. This should exacerbate interrogative suggestibility particularly in cases accompanied by a weak recollection of the event which is the reason for the interrogation [14–16].

### Intonation

As noted above, intonation is defined as “the variations which take place in the pitch of the voice in connected speech” ([2], p. 275). Intonation has traditionally been described either in the form of contours, i.e. as tonal movements in certain directions, or in the form of levels into which the vocal range of a speaker can be divided. Contour-based models (e. g. [17–21]) describe intonation in the form of dynamic tone contours (intonation progressions) and attach particular importance to the nucleus as the most prominent syllable of the utterance. Level models (e. g. [22–25]) consider intonation patterns to be sequences of different pitches. They describe pitches of certain structurally relevant syllables and postulate at least two (high [H] and low [L]), at most three (high [H], low [L], and mid [M]) levels for the description of intonation. Tones or tonal movements are understood as a sequence of target points that lie on these levels. On the one hand, tones serve to emphasize certain syllables; on the other hand, they can act as initial or final boundary markers for intonationally relevant phrases (e.g. [26]). The following explanations are based on the contour-based Kiel Intonation Model (KIM, [19]). The KIM was selected because prior research in legal psychology has corroborated its applicability to explain interrogative suggestibility ([4]).

### Intonation contours

**Fig 1:**
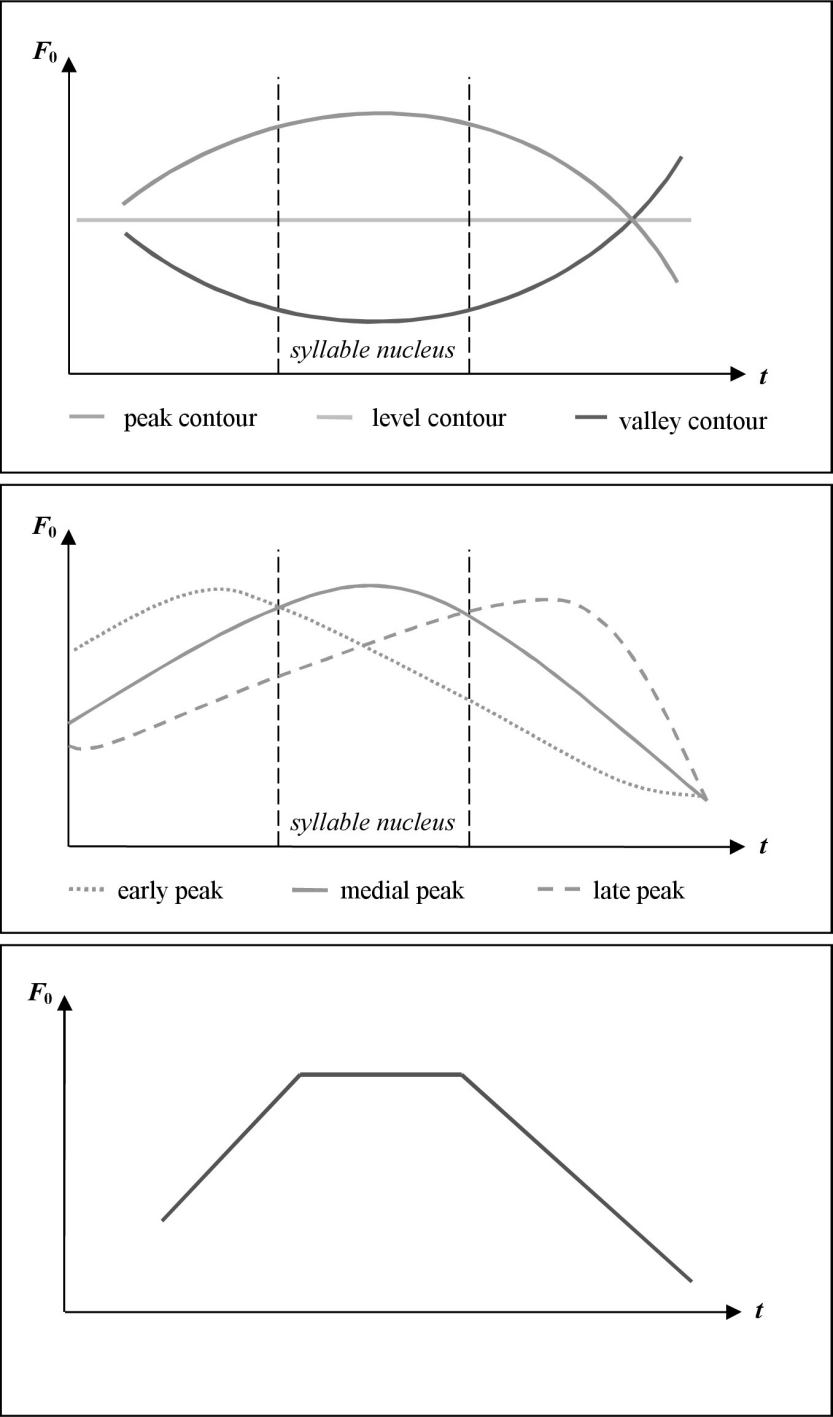
Schematic overview of *F*_0_ fundamental frequency contours.

In speech, an utterance can be divided into several segments–its prosodic phrases. On the basis of studies concerned with German intonational form-function relations of natural-utterance fundamental frequency (F_0_) contours, Kohler developed the KIM [19] that systematically labels and analyzes intonation structures specific to the German language. F_0_ has been defined “as the frequency of the sinusoid that evokes the same perceived pitch (residue pitch, virtual pitch, etc.) as the complex sound that represents the input speech signal” ([27]; p. 184). Hence, F_0_ is an acoustical parameter whereas pitch is a perceptual parameter. Kohler [19] differentiated peak, valley, or level contours (so-called basic contours; Fig 1) depending on the placement of the F_0_ maximum or minimum in relation to the vowel carrying the sentence stress in the center of the syllable (the syllable nucleus), whereby only peak level contours will be regarded in the following.

#### Perceptive interpretation of intonation contours

The most frequent intonation contour in German is the *peak contour* ([28]; Fig 1). Its phonetic realization displays a convex F_0_ movement reaching its local maximum in the area of the syllable nucleus. Kohler [19] differentiates between three peak contours:

The *early peak contour* is characterized by an F_0_ maximum placed before the syllable nucleus. The F_0_ movement of this peak contour features a fast rise to its local maximum and a slow fall within the syllable nucleus, which causes a falling pitch perception. An early peak is predominantly used when known facts are discussed with no particular emotional involvement [28].

The *medial peak contour* is characterized by an F_0_ maximum which is placed within the syllable nucleus. Being the most frequently-used peak contour, it indicates novel facts and beginning arguments [28, 29]. That is, in German as well as in English it is used for the discourse of new items (e. g. [30–32]). New items (or broad focus items) are those which are added to the common ground by speakers. For example, if an utterance such as “Anna was on HOLIDAY” (with the capitalization signaling the use of a medial peak accent) is produced, this is a general statement with no implied contrast.

Finally, the *late peak contour* is characterized by an F_0_ maximum placed behind the syllable nucleus. The F_0_ movement of this peak contour features a slow rise to its local maximum and a fast descent during the segments following the syllable nucleus. Especially in the case of naïve listeners, this causes a rising pitch perception. In German and in English the late peak contour is used on focused items ([30–32]). Focused items (or narrow focused items) are those which are presented in contrast to another item. For example, if an utterance such as “Anna was on HOLIDAY” (with the capitalization signaling the use of a late peak accent) is produced, the interpretation is that Anna is on holiday, specifically as opposed to “at work”. The late peak contour also serves to express particular emotional involvement and surprise [28].

Each basic contour can occur in prosodic phrases, which only have one sentence stress. The subsequently emerging contour above the phrase is seen to be a global melodic unit. In case the prosodic phrases have more than one sentence stress, they form combinations of a number of basic contours (so-called concatenation patterns), which build a complex melodic course above the phrase. The concatenation patterns build superordinate patterns and new functions evolve. Kohler [19] differentiates phonologically different concatenation patterns, which may be dipped or non-dipped. A frequently occurring intonation pattern in German language involves a rise and a fall pitch accent concatenated by a high plateau–the hat pattern (also referred to as bridge accent [25] or rise-fall contour [33]). It is defined “as a non-dipped sequence of two peak accents”([34]; p. 1). The hat pattern’s semantic function overlays the function of late and early peaks. For example, a hat pattern creates a wide focus above the accentuated components and puts the content elements side by side in an emotionally neutral as well as non-prejudgmental way. As it summarizes the accentuated components acoustically as well as in terms of content, it also has a bracketing function that facilitates auditory grouping of related verbal content.

#### Intonation and memory performance

A study series by Frankish [35] investigating the auditory grouping of verbal information corroborated that intonation can influence memory performance. He found the reproduction of a number sequence structured by an intonation contour in natural speech from memory to be more accurate than in case of a structure that is merely created by pauses or numbers that are presented in a monotone (i.e. ungrouped) fashion. Similar findings could be made with blocked (according to the nomenclature of level models: low [L]LL, high [H]HH, LLL) or hierarchically (according to the nomenclature of level models: low [L] mid [M] high [H], LMH, LMH) structured realizations of verbal information, which were also contrasted with monotone presentations with or without pauses. Frankish [35] concluded that predominantly pronounced pitch accents as well as pitch curves occurring at the prosodic borders seem to facilitate the reproduction. However, it is not apparent from his studies to which extent which features of intonation contours in natural speech determine memory performance. Particularly, he does not differentiate between the presence of deliberate emphases, which can be achieved with pitch accents, and the pitch curves at the ends of prosodic phrases.

In summary, two fundamental characteristics for spontaneous and reading speech can be derived from the functions of intonation: First, intonation structures what is said and, at the same time, connects the prosodic phrases in relation to each other. Second, intonation emphasizes newly introduced and important information as well as information that is contrary to a person’s expectations delineating it from already known, less important information and information being concordant with what a person is expecting. If intonation functions are applied consistently in an interrogation, the result is a specific speaking style expressing the pragmatic intention of the speaker (pragmatics is a subfield of linguistics that studies how the transmission of meaning depends not only on structural and linguistic knowledge [e.g. grammar, lexicon, etc.] of the speaker or listener, but also on the context of the utterance, any pre-existing knowledge about those involved, the inferred intent of the speaker, and other factors; pragmatics explains how language users are able to overcome apparent ambiguity, since meaning relies on the manner, place, time, etc. as well as the intonation of an utterance). Crucially, while reading out a text, a record of interrogation, or test instructions, the interrogator could, for example, try to symmetrically structure the content into thematic sections. To this end, she/he might apply intonation patterns which according to Frankish’s [35] aspect of structure focus on the sentence accents as an overall configuration and put the content-related elements side by side. Additionally, an interrogator could emphasize text details which seem important to her/him in an isolating fashion focusing on the independence of the sentence accents and juxtaposing the content-related elements. Similarly, Calhoun [36] presents another approach relating to the notion that intonation creates information structure where a crucial component is the amount of prosodic salience imparted on an item relative to its expected prosodic salience. Notably, this can be regarded as a (subtle) deviation from Grice’s cooperative principle in speech [37] where the interrogee in an interrogation might be led to suppose that the interrogator has a strong reason for adding particular prosodic salience to a specific detail such as, for example, that the interrogee had answered unsatisfactorily or that the relevance of a detail had been overseen. This interaction of a subtle emphasis and its interpretation, in turn, might increase (potentially on a rather automatic level of information processing) the suggestive potential of the prosodically emphasized details and the interrogative suggestibility of the interrogee.

#### Peripheral and central details and memory performance

In light of the findings of research on interrogative suggestibility in a legal psychology context, it seems to be important whether the abovementioned emphases are realized on central or peripheral details of the stimulus material. According to Christianson and Loftus [38], central details could be identified in terms of their centrality to the subject’s attention, rather than relevance to the plot. Therefore, central details would be those details associated with material central to subjective attention, independent of whether they are also associated with material central to the event’s plot. A series of studies have indicated that the emotional intensity of an event is a significant predictor for how vividly the event is recalled [39, 40]. To this end, it is thought that memory for central details of (particularly negative) emotional events is well retained, whereas memory for peripheral details is poorly recalled [41–45]. Some studies have found that, whereas memory for peripheral details seems to be diminished by high levels of arousal, memory for central details appears to be facilitated [46, 38, 47]. Lanciano and Curci [48] demonstrated, that after an emotional event, also peripheral details may be stored, and that memory of these details is influenced by the memory task adopted as individuals provided more peripheral details when they were asked to remember these in a probed recall than in a free recall task.

However, as noted above, in light of the findings of linguistic research individuals have better recall of items with late peak accents, regardless of their status as central or peripheral in the narrative ([30–32]).The production of items with a late peak accent renders these items not only acoustically salient, but also salient in the discourse structure, by marking them as focused. Taking into account the intonational function of assigning salience to verbal information pieces, it is conceivable that the general memory advantage in favor of central details could also be weakened if the peripheral details of the stimulus material are emphasized in an isolated fashion during their presentation and are consequently put more into the focus of the listener.

### Current study

As intonation plays a role during the recall phases *after suggestive interrogation* [4], we sought to explore a possible earlier impact of intonation on memory performance as early as in the encoding phase. To this end, we aimed to analyze intonation effects in two experiments: Experiment 1 focused on the recollection of the given information as a whole (i.e. general impact of intonation style on free recall) whereas Experiment 2 analyzed the recollection of specific content elements that were emphasized or not (i.e. interplay of different item and emphasis types). We expected in Experiment 1 that participants who were presented with a story in a symmetrically structuring speaking style (i.e. neutral intonation, without specifically emphasizing any details) can freely recall more details than participants who heard the story in an isolating-emphasizing speaking style (i.e. fully emphasizing every new bit of information). In Experiment 2, on a more fine-grained level, we hypothesized an interaction of item type (i.e. peripheral, neutral, and central details) with type of emphasis (i.e. emphasizing central or peripheral details). Specifically, we expected that the difference between correctly recalled central and peripheral details is larger in case of emphasized central items than for emphasized peripheral items as it should be generally easier to recall central than peripheral details.

## Materials and methods

In order to test our hypotheses, participants in both experiments heard a story that they had to recall immediately and after a delay of 50 minutes. The presented story differed in the tonal patterns of the representation. The experimental procedure was in accordance with the ethical standards on human experimentation of the institutional ethics committee. As the study involved no intervention/treatment/drug application nor any distressing or personally sensitive content and participants were not sampled from a vulnerable or clinical population no official ethics votum was required at the research institution. The whole procedure was in accordance with the Helsinki declaration and all of its amendments. Informed consent was signed before participation in the study. Participants were free to withdraw consent and terminate their participation at any time during the experiments.

### Participants

A total of 100 first semester undergraduate psychology students from the Christian Albrecht University of Kiel participated in both experiments (*n* = 25 randomly assigned participants in each experimental condition, *N* = 50 in each experiment). In total, the samples contained 82 female and 18 male students (see Table 1 for more details). Gender was independent of experimental condition in Experiment 1, but not in Experiment 2. Therefore, we statistically controlled for possible gender effects in all analyses in both experiments (although further control analyses revealed that all results followed the same patterns without this covariate and effect sizes did not change substantially). Ninety-six participants were of German origin, four came from other countries; the latter, however, had sufficiently good knowledge of the German language. The average age was *M* = 22.79 years (*SD* = 4.13). Age was independent from experimental condition in both Experiments (Table 1). Importantly, groups were equivalent on level of basic cognitive skills (i.e. memory performance, Table 1) as they did not differ on verbal or figural retentiveness [49].

**Table 1:** Overview of sample descriptives and mean differences as a function of experimental conditions.

	Experiment 1							Experiment 2						
	Neutral Emphasis (*n* = 25)		Full Emphasis (*n* = 25)					Central Emphasis (*n* = 25)		Peripheral Emphasis (*n* = 25)				
	*M*	*SD*	*M*	*SD*	*t*	*p*	φ*/d*	*M*	*SD*	*M*	*SD*	*t*	*p*	φ*/d*
Gender *n* female (%)^a^	23	(92)	21	(84)		.667	-.12	15	(60)	23	(92)		.018	.38
Age	22.1	3.4	23.6	5.6	1.20	.237	0.32	23.4	3.7	22.0	3.5	1.41	.164	-0.39
Verbal Memory^b^	9.4	1.0	9.1	1.3	< 1	.342	0.26	8.8	1.9	9.0	1.4	< 1	.564	0.12
Figural Memory^b^	10.4	2.1	9.8	2.3	1.04	.305	-0.27	10.1	2.8	9.7	2.5	< 1	.557	-0.15

Note.

^a^ = Fisher’s exact test

^b^ = raw scores.

### Measures

Verbal and figural retentiveness–as potential confounders–were assessed by two different scales (verbal and figural retentiveness task) from the revised Intelligence Structure Test [49], a frequently used German intelligence test battery. In order to gauge the potential influence of intonation during interrogation, participants’ memory performance needed to be ascertained. To this end, we employed the German version of the GSS-1, the forensically relevant version of the GSS (GGSS-1; [4]) consisting of a short story of a robbery broken down into 40 distinct items (Table 2). The Kiel University Institute of Phonetics and Digital Speech Processing produced a high-quality digital natural voice recording of four versions of the GGSS-1 story that differed with respect to their pitch curve and/or the auditive prominence of a few words but not in content.

**Table 2:** German translation (GGSS-1) of the GSS-1 story.

Anna *Thomsen*/ aus *Berlin*/ *Mitte*/ war in *Spanien*/ im Urlaub,/ als sie außerhalb ihres Hotels/ *angegriffen*/ und ihrer *Handtasche* beraubt wurde./ In der Handtasche befanden sich *Reiseschecks*/ im Wert von *70 €*/ und ihr *Reisepass*./ Sie *schrie* nach *Hilfe*/ und *versuchte* sich zu verteidigen,/ indem sie einem der Angreifer/ gegen das *Schienbein* trat./ Die Polizei traf bald ein/ und die Frau wurde zur *nächstgelegenen Polizeistation* gebracht,/ wo sie von *Kriminalkommissar*/ *Delgado*/ befragt wurde./ Die Frau berichtete, sie sei von *drei Männern* angegriffen worden,/ und beschrieb einen von ihnen als *asiatisch-aussehend*./ Die Männer seien *schlank*/ und *Anfang Zwanzig* gewesen./ Der Polizeibeamte zeigte sich von der Geschichte *berührt*/ und schlug ihr vor, die Deutsche *Botschaft* aufzusuchen./ Sechs Tage *später*/ *stellte* die Polizei die Handtasche der Frau *sicher*,/ der Inhalt dieser wurde aber *nicht* mehr gefunden./ *Drei Männer* wurden schließlich *angeklagt*,/ *zwei* der Männer wurden dann verurteilt/ und erhielten *Gefängnisstrafen*./ Einer von ihnen/ war wegen *ähnlicher* Delikte/ bereits vorbestraft./ Gemeinsam mit ihrem Mann/ *Simon*/ und zwei Freunden/ *kehrte* die Frau nach *Deutschland* zurück,/ blieb jedoch *ängstlich*, wenn sie sich *allein* außerhalb des Hauses aufhielt.

*Note*. The forward slashes represent the distinct elements of the story (i.e. GGSS-1 items). Words that were accentuated in the isolating-emphasizing (i.e. fully emphasized) speaking style are printed in italics.

#### Experiment 1 –General intonation style

In order to realize the different levels of the independent variable for our first hypothesis, the story was recorded with either a symmetrically structuring or an isolating-emphasizing intonation style.

#### Symmetrically structuring intonation style

**Fig 2:**
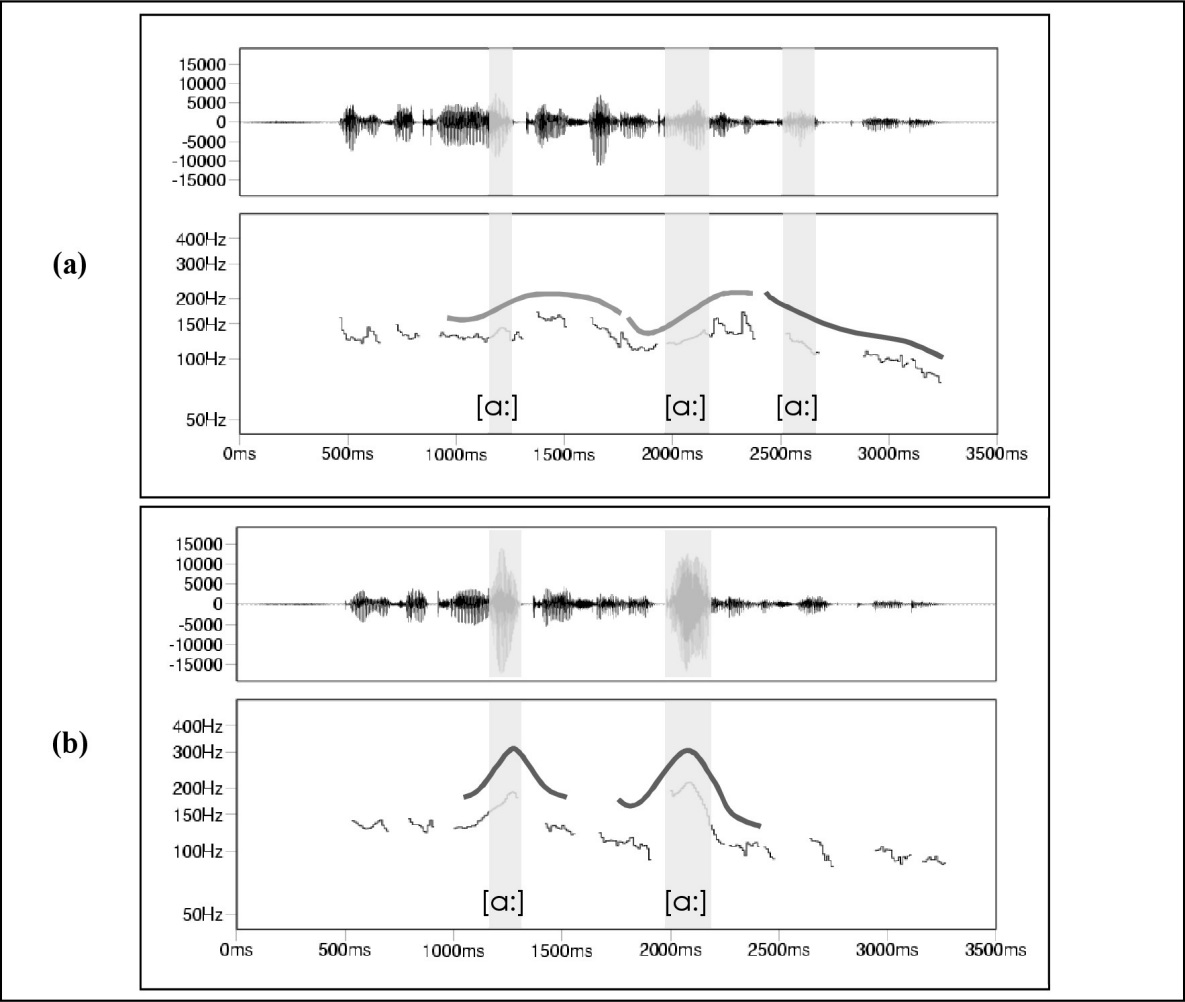
The partial sentence “…, wo sie von Kriminalkommissar Delgado befragt wurde” […, where she was interrogated by Detective Delgado], realized in (a) a symmetrically structuring speech style and (b) an isolating/emphasizing speech style. Above the *F*_0_ contour, there is a stylized visualization of its course. The shaded areas mark the vowels in the center of the syllables with sentence stress.

The first intonation variant is characterized by rising and falling pitch curves that follow each other and thus create an equilibrium of high and low tones at the ends of mutually dependent melodic units. This was realized by integrating successive peak contours in which the sentence stress elements are not focused individually, but as an overall configuration. As there is a notable interplay of late and early peaks when symmetrically structuring a text, hat patterns were formed predominantly out of these accent contours. Emphases of specific content elements were avoided entirely (see online supplement S1 File). Fig 2A illustrates the symmetrically structuring speaking style. In the following we refer to this experimental manipulation as the *neutral condition*.

#### Isolating-emphasizing intonation style

For the second intonation variant, all new as well as contrasting information elements were marked with noticeable accent contours whereas the connected background information was not specifically accentuated (see Table 2 for the accentuated parts of the GGSS-1). This speaking style is characterized by rising and falling pitch curves which reach their *F*_0_ maximum on the vowel in the center of the syllable that carries the sentence stress or a later syllable of the overall 40 isolated-emphasized details. It was realized with the help of successive peak contours in which the sentence stress elements are focused individually. Predominantly, medial and late peaks were concatenated, which–among other things–serve to signal novel facts. While medial peaks communicate the novelty of the information in a neutral way, late peaks express them with a special emotional involvement and surprise. Moreover, the auditive prominence of the accent-bound tonal patterns is relatively strong and partially shows signs of empathic accentuation (see online supplement S2 File). Fig 2B illustrates the isolating-emphasizing speaking style. In the following we refer to this experimental manipulation as the *fully emphasized condition*.

#### Experiment 2 –Type of emphasized details across item types

The stimulus material for the test of our second hypothesis consisted of the GGSS-1 story generally presented in a symmetrically structuring (i.e. neutral) intonation style. Within the story, however, we varied whether either six peripheral or central items were presented in an isolating-emphasizing intonation style (i.e. fully emphasized as described above). Hereby, peripheral and central items were distinguished from each other based on a pretest with 30 forensically naïve independent student raters who classified all GGSS-1 items story into each ten most peripheral or most central items (*ICC* across all items = .99, see Table 3 for items and rating frequencies).

**Table 3:** The six details of the GGSS-1 story most frequently rated as peripheral/central (percentage of highest ranking among 30 independent raters in brackets).

Peripheral details	Central details
Thomsen (93.3%)	außerhalb ihres Hotels [outside her hotel] (100%)
Mitte [subdistrict Mitte in Berlin] (93.3%)	70 € (100%)
nächstgelegene [nearest] (100%)	Anfang Zwanzig [early twenties] (100%)
Delgado (93.3%)	nicht mehr gefunden [were never found] (96.7%)
sechs Tage [six days] (93.3%)	Gefängnisstrafen [prison sentences] (96.7%)
Simon (96.7%)	ängstlich [frightened] (100%)

**Fig 3:**
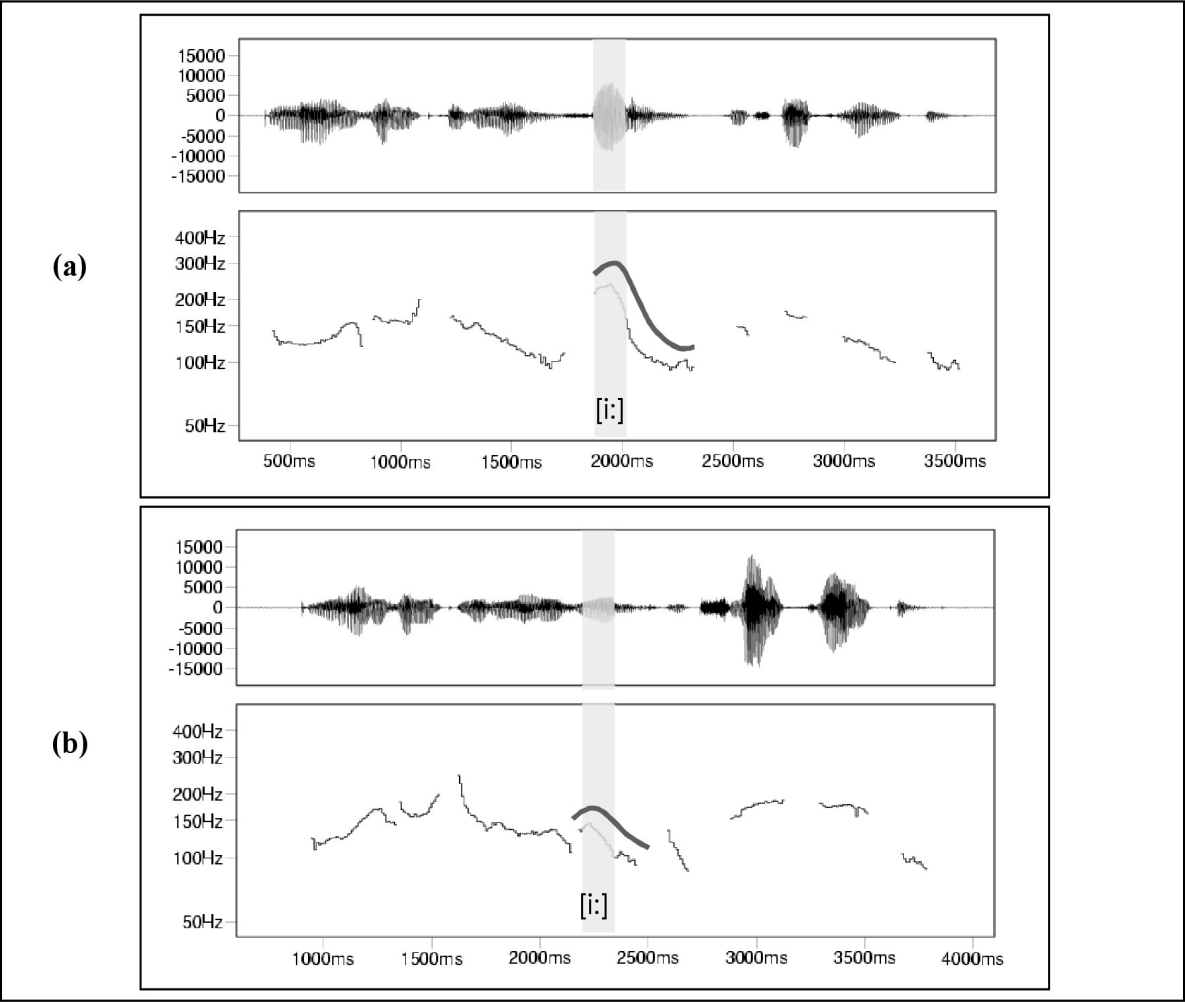
The detail “Simon,” realized with and without emphasis (a and b respectively). Above the *F*_0_ contour, there is a stylized visualization of its course. The shaded areas mark the vowels in the center of the syllables with sentence stress.

From these ratings we chose six items each that were most frequently rated as peripheral or central in order to guarantee a sufficiently strong contrast between the resulting GGSS-1 versions and the fully emphasized version described above. All 28 items that did not belong to the central or peripheral subset are referred to as neutral items in the following analyses. Fig 3 illustrates the intonation differences between the two versions (see online supplement S3 and S4 Files).

#### Dependent variables

In Experiment 1, our first hypothesis whether a fully emphasized compared to a neutral speaking style decreased the amount of freely recalled details was tested using correct immediate and delayed recall rates based on the 40 items of the GGSS-1 story. Immediate free recall was assessed directly after the presentation of the GGSS-1 story; delayed free recall was requested 50 minutes after the presentation of the respective story. Each recall measurement was based on participants’ self-written recounts of the memorized story rated by two independent raters (interrater reliabilities were excellent for all categories of dependent variables; *ICC*s > = .98, in case of non-congruent ratings an agreed on consensus rating was utilized for all statistical analyses). Each of the 40 distinct GGSS-1 items was scored 0 (item was not at all or wrongly reproduced), 0.5 (item was incompletely, ambiguously reproduced), and 1 (item was correctly reproduced).

The test of our second hypothesis concerning a putative interaction between type of item and type of item emphasis in Experiment 2 was based on the respective correct free recall rates for peripheral, neutral, and central item subsets as described above. Throughout all analyses mean correct free recall rates were used in order to ascertain comparable scores across the differing item subsets used for our hypothesis tests.

#### Procedure

The studies were conducted within groups of five participants in a soundproof research room at the former Kiel University Institute of Phonetics and Digital Speech Processing. After participants had been welcomed, they were asked for their written consent with regard to their study participation. Subsequently, the relevant GGSS-1 story variants were played back via a notebook with external loudspeakers. Next, participants wrote their immediate recall protocol. In the course of the following 50 minutes, participants worked on a number of filler tasks (e.g. intonation tests) unrelated to the present study. After that time period, the participants were asked to write their delayed recall protocol. Finally, the participants received certificates of participation for course credit and were dismissed. Test duration amounted to approximately 60 minutes.

## Results

Table 4 gives a descriptive overview of the focal dependent variables for both experiments.

**Table 4:** Descriptive overview of all dependent variables for Experiments 1 and 2.

Recall Rate	*M*	*SD*	Min	Max
Experiment 1				
Total *t*_1_	0.59	0.14	0.28	0.88
Total *t*_2_	0.58	0.14	0.31	0.89
Experiment 2				
Central *t*_1_	0.58	0.23	0.08	1.00
Neutral *t*_1_	0.58	0.14	0.30	0.89
Peripheral *t*_1_	0.57	0.22	0	1.00
Central *t*_2_	0.58	0.21	0.08	1.00
Neutral *t*_2_	0.58	0.14	0.34	0.86
Peripheral *t*_2_	0.56	0.23	0	1.00

*Note*. *N* = 50 for each Experiment.

### Experiment 1 –General intonation style

Our first hypothesis tested the effect of the general intonation style with which the GGSS-1 story was recorded. We conducted a 2 (Time: immediate vs. delayed recall) x 2 (Intonation Style: neutral vs. fully emphasized) mixed-model ANCOVA with Time varying within subjects and Intonation Style between participants. Participant Gender served as a covariate. The total correct free recall rate was the dependent variable (Table 5). The ANCOVA revealed a large impact of Participant Gender, *F*(1, 47) = 7.55, *p* = .008, η^2^ = .13, with male participants showing lower correct recall rates (notably, no further interaction with any other experimentally manipulated factor emerged). The expected main effect of Intonation Style was substantial as well, *F*(1, 47) = 7.10, *p* = .011, η^2^ = .13, showing higher reproduction rates in the neutral (*M* = 0.64, *SD* = 0.13) than in the fully emphasized condition (*M* = 0.53, *SD* = 0.13). Neither Time, *F*(1, 47) = 2.78, *p* = .102, η^2^ = .06, nor the Time x Speaking Style interaction, *F*(1, 47) < 1, η^2^ < .01, were statistically significant. Running the same analysis without the covariate yielded virtually the same effects with the exception that the main effect of Time became also significant, *F*(1, 47) = 4.95, *p* = .031, η^2^ = .09, indicating theoretically meaningful, generally reduced delayed recall rates across all participants.

**Table 5:** Correct recall rates for GGSS-1 items as a function of emphasis and item types across experiments.

	Experiment 1				Experiment 2			
Correct Recall Rate	Neutral Emphasis (*n* = 25)		Full Emphasis (*n* = 25)		Central Emphasis (*n* = 25)		Peripheral Emphasis (*n* = 25)	
	*M*	*SD*	*M*	*SD*	*M*	*SD*	*M*	*SD*
Total *t*_1_	0.65	0.13	0.54	0.14				
Total *t*_2_	0.63	0.13	0.53	0.13				
Peripheral *t*_1_					0.52	0.24	0.62	0.18
Neutral *t*_1_					0.59	0.16	0.58	0.13
Central *t*_1_					0.68	0.17	0.48	0.24
Peripheral *t*_2_					0.51	0.24	0.62	0.22
Neutral *t*_2_					0.58	0.16	0.58	0.13
Central *t*_2_					0.67	0.17	0.49	0.22

### Experiment 2 –Type of emphasized details across item types

**Fig 4:**
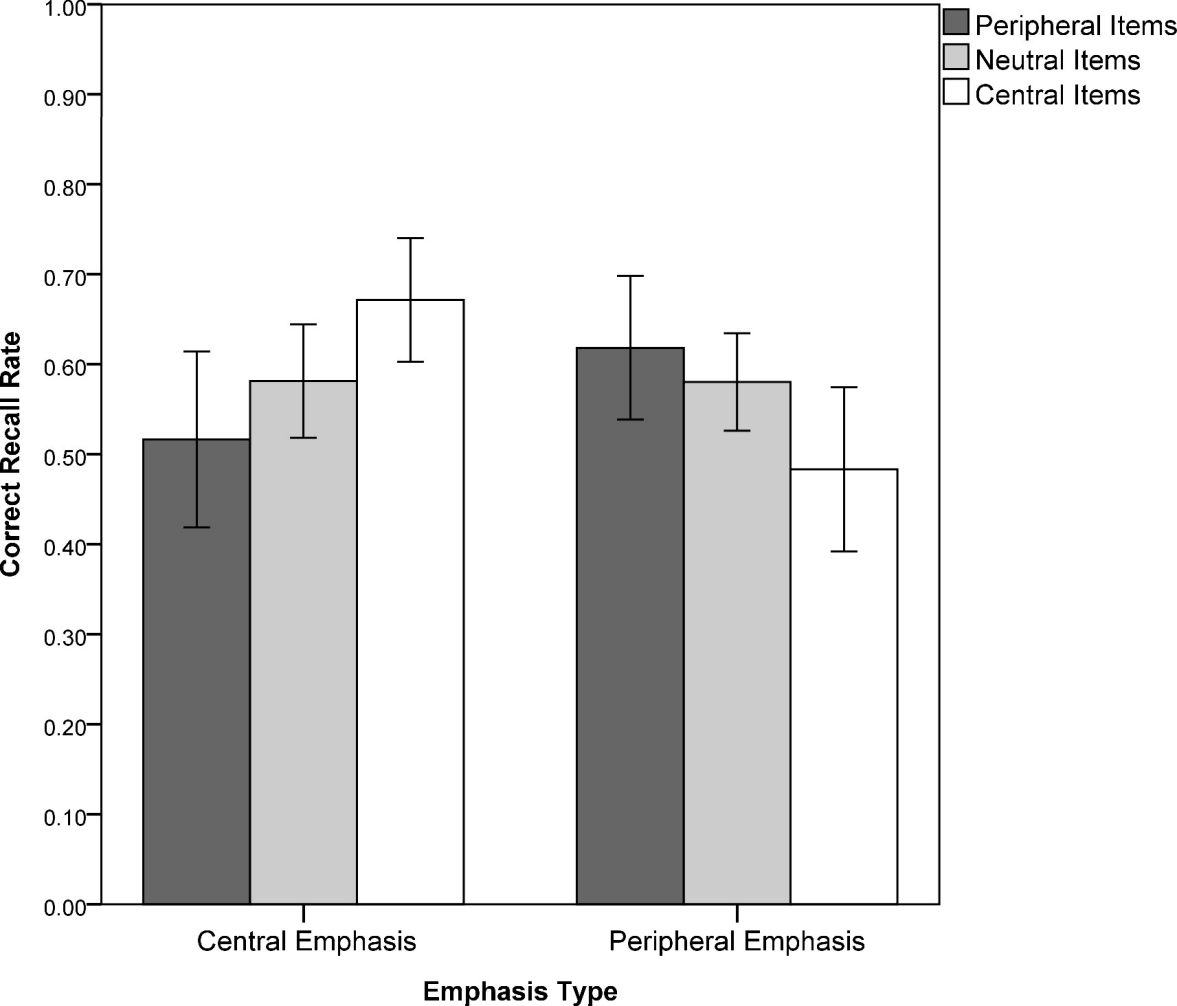
Mean recall rates across GGSS-1 item types as a function of emphasized details (error bars ± 95% confidence interval).

In order to test whether emphasizing specific item types impacted the correct reproduction rates for different item types we conducted a 2 (Time: immediate vs. delayed recall) x 3 (Item Type: peripheral vs. neutral vs. central items) x 2 (Emphasis Type: peripheral vs. central items emphasized) mixed-model ANCOVA with the first two factors varied within subjects and the last factor varied between participants and Participant Gender serving as covariate. Correct free recall rates for the three different item types served as the dependent variable (Table 5). This time, Participant Gender was not statistically significant, *F*(1, 47) = 3.08, *p* = .086, η^2^ = .06 (and, again, no further interaction with any experimental factor emerged). Running the same analysis without the covariate left the results virtually unaltered. We did not find any main effects of Time, *F*(1, 47) < 1, η^2^ = .02, Item Type, *F*(2, 46) = 2.99, *p* = .060, η^2^ = .12 (but note the expected large effect size driven by descriptively decreased recall rates for peripheral items), nor Emphasis Type, *F*(1, 47) = 1.78, *p* = .19, η^2^ = .04. Time was not further qualified by Item Type, *F*(1, 47) < 1, η^2^ = .04, nor Emphasis Type, *F*(1, 47) < 1, η^2^ < .01. Strikingly, as expected a substantial interaction of Item Type and Emphasis Type emerged, *F*(2, 46) = 10.55, *p* < .001, η^2^ = .31 (Fig 4). Post-hoc dependent sample *t*-tests revealed that in case of emphasized central items correct reproduction for central items (*M* = 0.67, *SD* = 0.17) was larger than for peripheral items (*M* = 0.52, *SD* = 0.24), *t*(24) = 3.07, *p* = .005; *d*_z_ = 0.75, whereas the opposite emerged in case of emphasized peripheral items with larger recall rates for peripheral items (*M* = 0.61, *SD* = 0.19) than for central items (*M* = 0.48, *SD* = 0.22), *t*(24) = 3.15, *p* = .004; *d*_z_ = 0.65. This pattern was not further qualified by interacting with Time, *F*(2, 46) < 1, η^2^ = .01.

## Discussion

### Intonation style

As proposed, participants who were presented the GGSS-1 story in a symmetrically structuring speaking style were able to deliver a more precise free recall than individuals who were presented the story in an isolating-emphasizing speaking style. This large effect can possibly be explained by the characteristic interplay of late and early peaks of the former intonation as it does not only cause a homogeneous connection of the content elements on a local level–on which its prosodic and semantic function has been examined so far [34, 50]–but also in the case of extensive utterance structures. In contrast, the multitude of content elements marked as novel as well as contrasting with at the same time reduced melodic grouping possibilities of the fully emphasized intonation seem to have influenced both information intake and information processing negatively. Here, text content might not have been perceived in its whole gestalt, but rather as an extended listing of numerous isolated details. This result is also concomitant with Calhoun’s [36] notion that the amount of prosodic salience imparted on an item relative to its expected prosodic salience emphasizes informational value. Thus, emphasizing every aspect of a story provides little information to the listener about what is important, leading to a less structured narrative that is more difficult to recall.

### Type of emphasized details across item types

Our second hypothesis that the difference between correctly recalled central and peripheral details is larger in case of emphasized central items than for emphasized peripheral items was confirmed. However, the difference between peripheral vs. central items was not only weakened but reversed. Thus, the influence of a story’s tonal pattern on memory performance is not equal for all kinds of information, but seems to be determined by whether certain item types are rendered salient during the encoding phase. Research on interrogative suggestibility has repeatedly shown memory performance advantages in favor of the central details of emotionally more or less stressful stimulus material [41–45]. However, in the present study adding a specific intonational emphasis–apart from emotional salience–increased the ability to recollect the selectively emphasized material. Strikingly, peripheral details thus can be shown to be better recalled than central items once they have been specifically emphasized via intonation during the encoding phase. The production of those items with late peak contours renders these items not only acoustically salient, but also salient in the discourse structure, by marking them as focused (e. g. it was Anna THOMSEN and not Anna Hansen that got robbed).This is a significant finding for forensic contexts as usually witness statements during veracity assessments are especially rated as credible if they contain a high amount of peripheral details [51, 52]. Thus, from an applied perspective, this becomes particularly problematic in judicial contexts where criteria-based content analysis [53] is regularly used to assess statement validity such as for example in German courts. In this paradigm recalling peripheral details is treated as a central indicator for statement veracity [54].

## Limitations

A number of limitations of the present study need to be acknowledged. First, participant gender (due to only a few male participants) was not well-balanced across both studies. However, although there has been a general main effect of decreased recall in the male subgroup in Experiment 1, Gender did not impact the focal intonation effects on memory encoding as revealed by further control analyses. More importantly, the influence of the tonal pattern was investigated solely within reading speech. It is open whether the effects can be transferred onto spontaneous speech. Furthermore, whether the functions for the described German prototypical intonation patterns similarly work in English must be analyzed in language related research taking into consideration the differential syntactic and pragmatic conditions of both languages. Moreover, both manipulated speaking styles should be understood as endpoints of a bipolar dimension either avoiding any emphasis or marking all novel and contrasting information with noticeable accent contours. Hence, intonation effects here are likely to be artificially inflated. Thus, to which extent the determined effects can be validated with other melodic realization remains an open empirical question. However, all these restrictions of the external validity dovetail with a strengthened internal validity as it was our primary aim to maximize chances to demonstrate intonation effects for more complex verbal material in the encoding phase for the first time at all. Finally, unlike in the standard GSS procedure, participants solely were asked to reproduce the GGSS-1 story without follow-up interviews that purposely introduce suggestive elements. We expect exacerbated interrogative suggestibility effects when intonation effects during the interrogation phase [4] are added on top of the intonation effects shown here for the encoding phase.

## Conclusion and implications

From an applied legal psychological perspective, this study underscores that intonation influences memory recollection to a significant degree. As our focal dependent variable (i.e. correct recall rate) was based on self-report assessments that did not involve any further interrogative interaction with an interviewer we can safely conclude that the experimental manipulations indeed impacted memory performance as early as in the encoding phase. Hence, our findings differentiate results from Frankish [35] by corroborating that the effects of intonation on memory performance can be allocated to several prosodic devices that differentially interact with information encoding as well as information processing. Although a symmetrically structuring speaking style generally promotes memory performance, it is highly unlikely that an interrogator treats all content elements of a text/statement that he reads out equally important. It is much more likely that some details are regarded as more important than others, for example due to (involuntary) confirmation bias [5], and hence are presented in an isolating-emphasizing fashion. This might lead to interrogation situations (or test instructions) where the listener’s memory is influenced in favor of the a priori interpretation of the assessor–a worst-case scenario in applied forensic contexts.

Given the impact of intonation effects, the results of the study at hand point to weaknesses with regard to the GSS’s implementation objectivity when used as an indicator for trait interrogative suggestibility. Since it is the interrogator’s choice to either read out the GSS story or to use a pre-recording, a systematic influence on test results in the memory recall part cannot be ruled out. To this end, the listener’s recollection quality at least partially depends on the speakers’ intonation through her/his use of emphases to highlight elements regarded as important when reading out the GSS story. Hence, it would be advisable to use a standardized pre-recorded version of the story *and* of the questions, where the former should be recorded in a symmetrically structuring speaking style and the latter with a phrase-final high pitch contour [4]. Of course these intonation effects might have conceivable (so far unexplored) implications for any memory performance measure that is based on free recall of non-standardized verbal material outside the forensic domain as well. Finally, future research should seek to disentangle the causal influences of interrogation conduct and intonation on interrogative suggestibility during encoding and recall phases as this might enhance our understanding of how to better avoid suggestive influences in forensic practice.

## Supporting information

S1 File Language file_symmetrically structuring speaking style. (WAV)

S2 File Language file_isolating-emphasizing speaking style. (WAV)

S3 File Language file_central emphasized story details. (WAV)

S4 File Language file_peripheral emphasized story details. (WAV)

S5 File Data set. (SAV)
